# Effect of systemic hypertension on right ventricular morphology and function: an echocardiographic study

**DOI:** 10.5830/CVJA-2010-013

**Published:** 2010

**Authors:** AA Akintunde, PO Akinwusi, OG Opadijo, OB Familoni

**Affiliations:** Division of Cardiology, Department of Internal Medicine, LAUTECH Teaching Hospital, Osogbo, Nigeria; Cardiology Clinic, Department of Internal Medicine III , Eberhard Karls University, Tubingen, Germany; Division of Cardiology, Department of Internal Medicine, LAUTECH Teaching Hospital, Osogbo, Nigeria; Division of Cardiology, Department of Internal Medicine, LAUTECH Teaching Hospital, Osogbo, Nigeria; Division of Cardiology, Department of Internal Medicine, Olabisi Onabanjo University Teaching Hospital, Sagamu, Nigeria

**Keywords:** hypertension, right ventricular function, echocardiography, systolic dysfunction, diastolic dysfunction

## Abstract

**Background:**

Hypertension is an important cardiovascular risk factor worldwide. It is associated with left ventricular hypertrophy (LVH). Both diastolic and systolic dysfunction may occur in hypertensive heart disease. The ventricles are structurally and functionally interdependent on each other. This was an echocardiographic study intended to describe the impact of left ventricular pressure overload and hypertrophy due to hypertension on right ventricular morphology and function.

**Methods:**

One hundred subjects with systemic hypertension and 50 age- and gender-matched normotensive control subjects were used for this study. Two-dimensional (2-D), M-mode and Doppler echocardiographic studies were done to evaluate the structure and function of both ventricles. Data analysis was done using the SPSS 16.0 (Chicago, Ill). Statistical significance was taken as *p* < 0.05.

**Results:**

Age and gender were comparable between the two groups. Hypertensive subjects had significantly increased left ventricular end-diastolic dimensions, posterior wall thickness, interventricular septal thickness, left atrial dimensions and left ventricular mass and index. The mitral valve E/A ratio was reduced among hypertensive subjects when compared to normal controls (1.15 ± 0.75 vs 1.44 ± 0.31, respectively; *p* < 0.05). A similar pattern was found in the tricuspid E/A ratio (1.14 ± 0.36 vs 1.29 ± 0.30, respectively; *p* < 0.05). Hypertensive subjects also had reduced right ventricular internal dimensions (20.7 ± 8.0 vs 23.1 ± 3.1 mm, respectively; *p* < 0.001) but similar peak pulmonary systolic velocity. The mitral e/a ratio correlated well with the tricuspid e/a ratio.

**Conclusion:**

Systemic hypertension is associated with right ventricular morphological and functional abnormalities. Right ventricular diastolic dysfunction may be an early clue to hypertensive heart disease.

## Summary

Hypertension is an important non-communicable disease in Africa. It is the most important cardiovascular risk factor.^1^-^4^ Left ventricular hypertrophy is often associated with hypertension and is an adaptive mechanism to maintain or normalise wall stress, sometimes at the expense of diastolic and long-axis systolic function.^5^ This adaptive mechanism is associated with changes in left ventricular parameters and in chamber dimensions, geometry and function.^6^,^7^ These changes are progressive and can ultimately lead to heart failure with systolic and/or diastolic dysfunction.^8^ However, the human heart functions as a unit that includes the right ventricle.^8^,^9^ The spectrum of changes in structure, function and shape of the left ventricle ultimately has an effect on the structure and function of the right ventricle.

The right ventricle is a thin-walled, low-pressure system. Studies that evaluated left ventricular function abound in the literature. However, studies about right ventricular structure and function among hypertensive subjects are rare. The aim of this study was to evaluate if any possible morphological and/or functional changes might occur in the right ventricle in subjects with systemic hypertension.

Echocardiography is a very useful and non-invasive diagnostic tool, which can be used to diagnose ventricular hypertrophy and various flow and pressure parameters in all cardiac chambers. Echocardiography in the evaluation of right ventricular function is important, as right ventricular dysfunction has been shown to correlate significantly with disease progression in subjects with chronic obstructive pulmonary disease,^10^ dilated cardiomyopathy^11^ and secondary pulmonary hypertension.^12^ Therefore, we proposed that it is important to document any possible right ventricular systolic and/or diastolic dysfunction in subjects with systemic hypertension.

This study was an echocardiographic evaluation of right ventricular systolic and diastolic function in subjects with systemic hypertension.

## Methods

The study group consisted of 100 adult Nigerian hypertensive subjects. Fifty age- and gender-matched normotensive subjects were recruited as controls. Hypertension was diagnosed using standardised criteria^13^ and was defined as the use of antihypertensive therapy or the persistent elevation of blood pressure above 140/90 mmHg on two or more occasions with the patient in a sitting position for at least five minutes. Patients were subjected to history taking, physical examination and simple clinical investigations. Demographic parameters, including age, gender, occupation and associated symptoms were obtained. The weight was taken in light clothing to the nearest 0.5 kg, while the height was taken to the nearest 1 cm using a stadiometer.

## Echocardiography

All subjects had echocardiography performed on them with the use of a SUIS APOGEE ultrasonograph with a 3.5-MHz transducer. Echocardiography was performed according to the recommendations of the American Society of Echocardiography (ASE).^14^ The patients were placed in the left lateral decubitus position. The left parasternal view was used to take the following measurements: right ventricular wall dimension and right ventricular end-diastolic diameter, interventricular septal diameter (IVSD), LV end-diastolic diameter (LVEDD), posterior wall diameter (PWD) and left atrial dimension (LAD).

LV mass was calculated using the Devereux formula^15^ and the LVMI was derived. Left ventricular hypertrophy was defined as a LVMI above 125 g/m^2^ for both genders, as used in the Framingham study for the evaluation of LVH in subjects with hypertension.^16^ The left ventricular ejection fraction was calculated using the M-mode measurements of the left ventricle.

The pulse Doppler studies were reported from the apical four chambers or alternatively the parasternal short-axis view. The Doppler sample volume was placed at the level of the tricuspid annulus and recordings from three cycles were averaged. The following measurements were taken: peak early E wave, peak late atrial A diastolic velocities and velocities time intervals. At the right ventricular outflow tract, the maximum and mean pulmonary systolic pressures were obtained. Similar measurements were made at the mitral valve annulus and at the left ventricular outflow tract. All the recordings were obtained for three cycles and their averages were used for calculation. All measurements were taken with the subjects in quiet respiration.

## Statistical analysis

Data were analysed using the Statistical Package for Social Sciences SPSS 16.0, (Chicago, Ill). Quantitative data were summarised using means ± standard deviation, while qualitative data were summarised using percentages and proportions. Comparisons between groups were made using independent *t*-test and chi-square as appropriate. Correlation analysis was done for some diastolic and systolic parameters. Statistical significance was taken as *p* < 0.05.

Table 1

**Table 1.** Demographic And Clinical Parameters Of Study Population

*Variable*	*Hypertensives (n = 100)*	*Controls (n = 50)*	p*-value*
Mean age (years)	57.5 ± 13.33	55.9 ± 11.33	0.12
Mean SBP (mmHg)	158.8 ± 18.1	117.8 ± 10.7	< 0.001**
Mean DBP (mmHg)	92.9 ± 13.1	76.6 ± 8.5	< 0.001**
Gender (M/F)	65/35 (65/35%)	28/22 (56/44%)	0.404
Mean BMI (kg/m^2^)	27.6 ± 5.9	24.8 ± 4.5	0.035
Mean BSA (/m^2^)	1.8 ± 0.2	1.7 ± 0.2	0.19
Mean PP (mmHg)	59.51 ± 17.9	41.1 ± 9.3	< 0.001**

**Statistically significant. SBP: systolic blood pressure, DBP: diastolic blood pressure, M: males, F: females, BMI: body mass index, BSA: body surface area, PP: pulse pressure.

## Results

The hypertensive subjects were well matched in age and gender (*p* > 0.05). The mean age of the hypertensive subjects was 57.5 ± 13.3 years while the mean age of the control subjects was 55.9 ± 11.3 years (*p* > 0.05). The gender distribution was also similar between the two groups. As expected, the systolic blood pressure, diastolic blood pressure and pulse pressure were significantly higher among the hypertensive subjects than the controls (158.8 ± 18.1, 92.9 ± 13.1, 59.51 ± 17.9 vs 117.8 ± 10.7 mmHg, 76.6 ± 8.5 mmHg, 41.1 ± 4.5 mmHg, respectively; *p* < 0.001). The hypertensive subjects also had a higher body mass index than the controls (27.5 ± 5.9 vs 24.8 ± 4.5 kg/m^2^, respectively; *p* < 0.05)

As shown in Table 2, the echocardiographic parameters show significant variations between the hypertensive subjects. The left ventricular chamber and wall dimensions were significantly higher among the hypertensive subjects than the controls. However, the right ventricular chamber dimensions were higher in the controls than the hypertensive subjects (23.1 ± 3.1 vs 20.7 ± 8.0 mm, respectively; *p* < 0.001).

**Table 2.** Echocardiographic Parameters Of Study Participants

*Variable*	*Hypertensives*	*Controls*	p*-value*
Mean IVSD (mm)	12.2 ± 2.6	10.2 ± 0.7	< 0.001**
Mean LVEDD (mm)	47.3 ± 7.6	45.0 ± 5.2	< 0.001**
Mean PWT (mm)	11.8 ± 2.5	9.6 ± 1.3	< 0.001**
Mean RVD (mm)	20.7 ± 8.0	23.1 ± 3.1	< 0.001**
Mean AOD (mm)	30.6 ± 4.8	32.5 ± 3.3	< 0.001**
Mean LAD (mm)	37.0 ± 8.1	30.8 ± 4.1	< 0.001**
Mean mitral E (m/s)	0.62 ± 0.22	0.62 ± 0.27	< 0.05*
Mean mitral A (m/s)	0.68 ± 0.17	0.54 ± 0.35	< 0.05*
Mean mitral E/A	0.91 ± 0.75	1.24 ± 0.31	0.035*
Mean DT (msec)	209.0 ± 54.7	148.0 ± 27.9	< 0.001**
Mean IVRT	105.4 ± 28.4	82.0 ± 9.6	0.072
Mean TE (m/s)	0.39 ± 0.12	0.46 ± 0.16	0.022**
Mean TA (m/s)	0.44 ± 0.11	0.38 ± 0.11	0.032**
Mean peak pulm vel (m/s)	0.61 ± 0.16	0.80 ± 0.2	0.442
Mean pulm vel (m/s)	0.61 ± 0.16	0.64 ± 0.26	0.36
Mean tricuspid E/A	0.89 ± 0.36	1.29 ± 0.30	0.04**
Mean FS (%)	46.6 ± 7.6	39.1 ± 5.2	< 0.001**
Mean LVMI (g/m^2^)	121.1 ± 35.9	87.6 ± 15.5	< 0.001**

**Statistically significant. IVSD: interventricular septal dimension in diastole, LVEDD: left ventricular end-diastolic dimension, PWT: posterior wall thickness in diastole, RVD: right ventricular diastolic dimension, AOD: aortic root dimension, LAD: left atrial dimension, Mitral E/A: mitral E/A ratio, DT: deceleration time, IVRT: isovolumic relaxation time, TE: tricuspid E (early) wave velocity, TA: tricuspid A (late atrial) velocity, Peak pulm vel: peak pulmonary systolic velocity, Mean pulm vel: mean pulmonary velocity, Tricuspid E/A: tricuspid E/A ratio, FS: fractional shortening, LVMI: left ventricular mass index.

The pulsed Doppler echocardiographic measurements are shown in Table 2. The pulmonary systolic velocities were similar among the hypertensive subjects and the controls (0.89 ± 0.25, 0.61 ± 0.16 vs 0.80 ± 0.2, 0.64 ± 0.26 m/s, respectively; *p* > 0.05). However, the tricuspid early wave velocity was lower among the hypertensive subjects than among the controls (0.39 ± 0.12 vs 0.46 ± 0.16 m/s, respectively; *p* < 0.05) while the peak atrial velocity was higher among the hypertensive subjects, 0.44 ± 0.11 vs 0.38 ± 0.11 m/s, respectively; *p* < 0.05) (Tables 3, 4).

**Table 3.** Gender And Doppler Echocardiographic Parameters Between Hypertensives With LVH And Those Without

*Variable*	*Hypertensives with LVH (n = 40)*	*Hypertensives without LVH (n = 60)*	p*-value*
Gender (M/F)	31/9	34/26	0.032**
Tricuspid E/A	1.21 ± 0.41	1.1 ± 0.31	0.187
Mean pulm vel (m/s)	0.58 ± 0.14	0.63 ± 0.16	0.171
Mean peak pulm vel (m/s)	0.86 ± 0.21	0.92 ± 0.27	0.266
TE (m/s)	0.38 ± 0.11	0.43 ± 0.13	0.064
TA (m/s)	0.42 ± 0.10	0.46 ± 0.12	0.09
LAD (mm)	39.5 ± 8.8	35.3 ± 7.2	0.01**
RVD (mm)	21.5 ± 7.8	20.0 ± 8.1	0.379

**Statistically significant. LVH: left ventricular hypertrophy, TE: tricuspid E (early) wave velocity, TA: tricuspid A (late atrial) velocity, Peak pulm vel: peak pulmonary systolic velocity, Mean pulm vel: mean pulmonary velocity, Tricuspid E/A: tricuspid E/A ratio, RVD: right ventricular dimension, LAD: left atrial dimension.

**Table 4.** Pattern Of Left Ventricular And Diastolic Dysfunction Among Treated Hypertensives

*Class of diastolic dysfunction*	*Left ventricle (n = 100)*	*Right ventricle (n = 100)*
Normal	18	45
Reversed	52	39
Pseudonormal	20	9
Restrictive	10	7

**Fig. 1.**
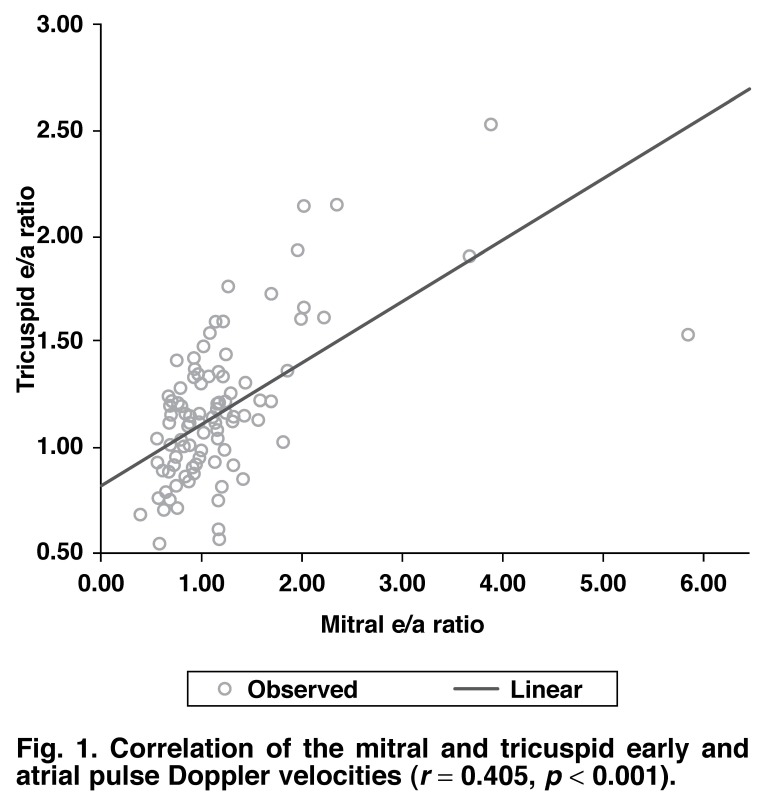
Correlation of the mitral and tricuspid early and atrial pulse Doppler velocities (*r* = 0.405, *p* < 0.001).

**Fig. 2.**
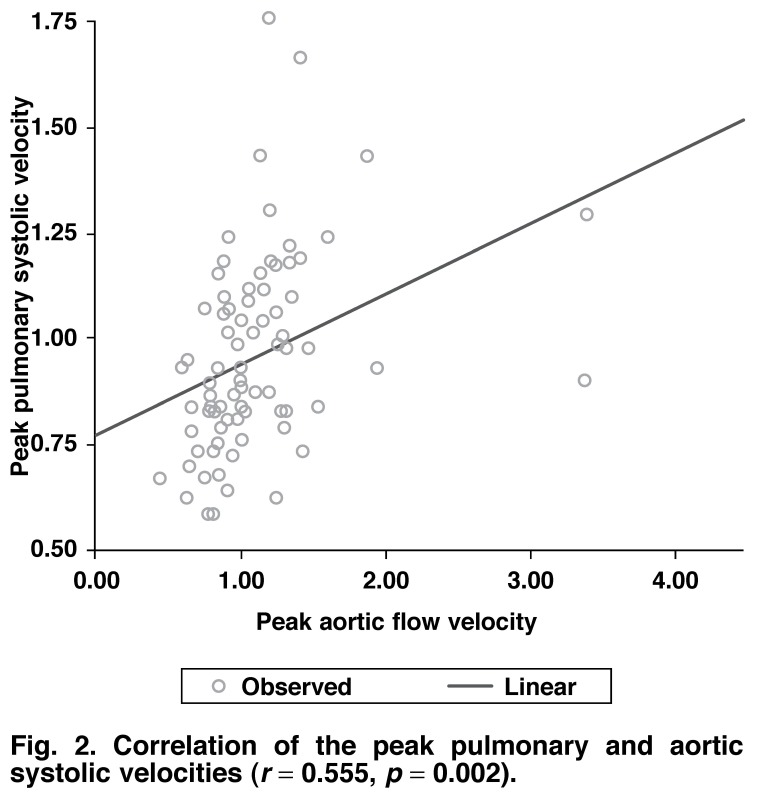
Correlation of the peak pulmonary and aortic systolic velocities (*r* = 0.555, *p* = 0.002).

A significant statistical correlation was found between the mitral E/A ratio and the tricuspid E/A ratio. A similar association was also demonstrated between the peak pulmonary systolic velocity and the peak aortic systolic velocities (Figs 1, 2).

## Discussion

This study demonstrated that right ventricular diastolic dysfunction accompanies left ventricular diastolic dysfunction that is due to hypertensive heart disease. This is in agreement with similar studies done among Caucasian hypertensive subjects.^17^,^18^ Right ventricular structural changes also occur in hypertensive heart disease.

In this study, the interventricular septal and posterior wall thickness, common to both ventricles were significantly thicker among the hypertensive subjects than in the controls. Left ventricular end-diastolic dimensions were higher among the hypertensive subjects than in the controls, while right ventricular dimensions in diastole were lower among the hypertensive subjects. Nunez *et al*.^19^ has demonstrated that right ventricular wall hypertrophy occurs in hypertensive subjects. It can be deduced that the combination of increased thickness of the posterior wall, interventricular septal dimension and right ventricular wall thickness will ultimately lead to at least progressive reduction in the right ventricular end-diastolic dimensions before progressive dilatation may occur in the right heart. The presence of LVH may also further lead to a compromise of the right ventricular internal dimension due to an increase in wall thickness.

An increased distension of either ventricle during diastole has been shown to alter the compliance and geometry of the opposite ventricle by either a direct mechanical effect (displacement of the septum) or some other indirect process.^20^ The right and left ventricles share common muscle bundles, septum and pericardium.^20^-^22^ The mechanisms of ventricular interaction are unknown but may relate to restriction of ventricular filling by the pericardium,^23^ although most work has assessed only the effect of RV volume expansion on LV function,^24^ rather than vice versa. This suggests that hypertension and left ventricular hypertrophy influence both ventricles. Right ventricular hypertrophy has been demonstrated among subjects with systemic hypertension.^17^-^19^

This study also demonstrated significant differences in the pulse wave velocities between hypertensive and normotensive subjects, which suggest that right ventricular diastolic dysfunction occurs in association with left ventricular diastolic dysfunction among these subjects. Several studies have shown that left ventricular diastolic dysfunction occurs early in hypertension.^17^,^19^,^24^,^25^ Left ventricular diastolic dysfunction is associated with the reduction of the early E wave and the subsequent increase in the amplitude of the atrial A wave. This may then be followed by several changes. Diastolic dysfunction has been divided into four groups depending on various parameters (E/A ratio, isovolumic relaxation time and deceleration time): early stages, pseudonormalisation, late and restrictive pattern of diastolic dysfunction.^26^

Doppler studies of cardiac flow and tissue movement remain the gold standard for estimating diastolic dysfunction with echocardiography.^26^,^27^ Doppler mitral flow pattern is very useful in estimating diastolic dysfunction and by itself is usually adequate to identify grade I (abnormal relaxation) and grade III (restrictive filling) diastolic dysfunction. The remaining subjects can be categorised as normal diastolic function or as having pseudonormalisation (grade II diastolic dysfunction) by additional testing such as the Valsalva manoeuvre, pulmonary venous flow pattern or tissue Doppler studies.

The abnormal relaxation that is frequently associated with ageing has been suggested to be due to the associated myocardial abnormalities and increased presence of cardiovascular risk factors and may therefore probably not constitute physiological aging.^28^ Although the right ventricle is a lower pressure system and echocardiographic assessment may be more difficult than the left ventricle due to its shape and morphology, interventricular dependence suggests that similar changes may occur in the right ventricle to those in the left ventricle due to the trophic effect of systemic hypertension and volume changes in the left ventricle.

This study demonstrated a similar pattern of tricuspid wave velocities in diastole and mitral wave velocities with good statistical correlations (*r* = 0.405, *p* < 0.001, Fig. 1). Also, the peak aortic and peak pulmonary systolic velocities showed similar statistical correlations (*r* = 0.555, *p* < 0.05, Fig 2). This suggests furthermore that right ventricular function is affected by hypertension in a similar pattern to left ventricular function. These changes may be subtle in the early stages of the disease and may require the use of more specific diagnostic parameters such as tissue Doppler imaging and strain echocardiography.^27^,^29^

However, this study failed to show a statistically significant difference in the mean pulmonary systolic and peak pulmonary systolic velocities between hypertensive subjects and controls. We suggest that the changes in the pressures of the right ventricle, although it is a low-pressure system, is influenced by the pressure and volume changes associated with hypertension. There is however no evidence that the right ventricular changes occur at the same time as those of the left ventricle. Prospective studies could document the timing of the right ventricular changes associated with hypertension.

## Conclusion

Hypertension affects the diastolic function of the left ventricle and these changes are accompanied by similar changes in the right ventricle. Whether these right heart changes occur early, at the same time as left heart changes due to the interdependence of the two structures, or whether it is a secondary phenomenon possibly related to pulmonary vascular changes remains to be proven by further studies. These changes occur in both the left and right ventricles. The changes are also prominent in the diastolic wave velocities, right ventricular wall dimensions and internal chamber dimensions.
